# A Highly Sensitive Electrochemical DNA Biosensor from Acrylic-Gold Nano-composite for the Determination of Arowana Fish Gender

**DOI:** 10.1186/s11671-017-2254-y

**Published:** 2017-08-10

**Authors:** Mahbubur Rahman, Lee Yook Heng, Dedi Futra, Chew Poh Chiang, Zulkafli A. Rashid, Tan Ling Ling

**Affiliations:** 1 Department of General Educational Development (GED), Faculty of Science & Information Technology, Daffodil International University, Dhanmondi, Dhaka, 1207 Bangladesh; 2 School of Chemical Sciences and Food Technology, Faculty of Science and Technology, Universiti Kebangsaan Malaysia, 43600 UKM Bangi, Selangor Darul Ehsan Malaysia; 3 Southeast Asia Disaster Prevention Research Initiative (SEADPRI-UKM), Institute for Environment and Development (LESTARI), Universiti Kebangsaan Malaysia, 43600 UKM Bangi, Selangor Darul Ehsan Malaysia; 4 Freshwater Fisheries Research Division, FRI Glami Lemi, 71650 Jelebu, Titi, Negeri Sembilan Darul Khusus Malaysia; 5 The Department of Chemistry Education, Faculty of Education, Universitas Riau, Pekanbaru, Riau 28293 Indonesia

**Keywords:** DNA biosensor, Electrochemical biosensor, Acrylic microspheres, DNA hybridization, Photopolymerization, Arowana DNA

## Abstract

The present research describes a simple method for the identification of the gender of arowana fish (*Scleropages formosus*). The DNA biosensor was able to detect specific DNA sequence at extremely low level down to atto M regimes. An electrochemical DNA biosensor based on acrylic microsphere-gold nanoparticle (AcMP-AuNP) hybrid composite was fabricated. Hydrophobic poly(n-butylacrylate-N-acryloxysuccinimide) microspheres were synthesised with a facile and well-established one-step photopolymerization procedure and physically adsorbed on the AuNPs at the surface of a carbon screen printed electrode (SPE). The DNA biosensor was constructed simply by grafting an aminated DNA probe on the succinimide functionalised AcMPs via a strong covalent attachment. DNA hybridisation response was determined by differential pulse voltammetry (DPV) technique using anthraquinone monosulphonic acid redox probe as an electroactive oligonucleotide label (Table 1). A low detection limit at 1.0 × 10^−18^ M with a wide linear calibration range of 1.0 × 10^−18^ to 1.0 × 10^−8^ M (*R* ^2^ = 0.99) can be achieved by the proposed DNA biosensor under optimal conditions. Electrochemical detection of arowana DNA can be completed within 1 hour. Due to its small size and light weight, the developed DNA biosensor holds high promise for the development of functional kit for fish culture usage.

## Background

Asiatic arowana (*Scleropages formoss*), a freshwater fish, [1] is widely distributed over the countryside of Southeast Asia region such as Malaysia, Singapore, Thailand, Indonesia, Cambodia, Vietnam, Laos, Myanmar and the Philippines. In addition, the arowana fish is also found in Australia and New Guinea [1–4]. It is popularly known as dragonfish, Asia bonytongue, kelisa, or baju-rantai [5, 6]. It is still surviving as a primitive fish species from the Jurassic era [7, 8]. The Chinese and Asian people considered it as a symbol of good luck and happiness, along with many other cultures [6]. Generally, the arowana is around 7 kg weight and 1 m long in their mature age [9]. This ornamental fish possesses attractive colours and morphology and can be identified by its distinctive physical features, such as comparatively long in body size, a large pectoral fin, and the dorsal and anal fins are positioned far back on the body. There are three main colour varieties, i.e. golden, red, and green of closely related freshwater fish within the Asian arowana species. There are also several other distinct species derived from different parts of the Southeast Asia and are regional to many river systems [8].

Due to its high popularity and great demand in ornamental purposes, Asian arowana has been fiercely hunted for profits [6], and results in a rapid decline of its population. Considering its high demand in ornamental industry, the over-exploitation of natural populations, and the rarity of natural habitats due to changes in the living environment, Asian arowana has been classified as an endangered species threatened with extinction since 1980 by the Convention on International Trade in Endangered Species of Wild Fauna and Flora (CITES) and has recently listed as endangered by the 2006 IUCN Red List [1, 3, 8, 10, 11]. However, the commercial trading of this endangered species is prohibited under CITES except in certain countries, e.g., Indonesia, Singapore, and Malaysia. [2, 3, 12]. There are a number of CITES registered cultivators in Asia actively carrying out farming and trading of arowana fish [2, 12]. This Asian freshwater fish consists of geographically isolated strains, and it is the only member of the species with different colour varieties that is based on different geographic distributions throughout the rivers of Southeast Asia. The species distribution is now far more widespread, which extends to the Nile River of Africa, the Amazon River of South America, Australia, and New Guinea [1, 4, 8].

Among the different colours of Asiatic arowana, red and golden arowana fishes are the most expensive and popular ornamental pets in the hatchery industry compared to black, green, silver, and others colour varieties [1, 5, 10, 13]. The egg thievery phenomenon of Asiatic arowana is atypical compared to other fish species. In general, arowana fishes get mature at the age of 3–4 years, and they lay only a few eggs (30–100) [14, 15] of extra-large size (around 1 cm in diameter) [16]. Interestingly, the fertilised eggs and larvae are then protected and grown up in the mouth of male arowana fishes, and they show high parental care. To identify the gender based on visual observation of the baby arowana is difficult because there is no distinctive phenotypic organ of sexual dimorphism [14, 15]. Only one of the parents (presume to be the male) can be identified as the offspring are harvested from his mouth. The other parent cannot be identified from among a number of potential parents [16].

Usually, the hobbyists keep the baby arowana fish for their ornamental purposes in the aquarium as well as for cultivation in the fish farm. However, all types of juvenile arowana fishes are sold at the same price, because of the lack of assistive technology for gender and colour variety differentiation. Until the present time, there is no established method published to identify the gender and colour of arowana fishes at their juvenile stage. Instead, hundreds of studies have been carried out using DNA analysis based on genetic structure and biography of arowana fishes in the attempt to identify the gender and colour at their early age. Traditional method based on body size and mouth cavity estimations can only be made at around 3 months of age of baby arowana for gender and colour identifications [17]. However, this conventional visual examination method is time-consuming and often provides inaccurate result. On the other hand, the widely used standard methods based on DNA sequencing, i.e., polymerase chain reaction (PCR) and gel electrophoresis are labour-, time-, and resource-demanding. An alternative algorithm of inventive problem solving (ARIZ) method was previously employed for the detection of arowana gender detection [18]. ARIZ is an alternative tool for gender detection, containing nine different parts and a total of 40 complex steps. It requires a very long time to learn and practice and demands highly experienced personnel to operate. For example, the application of ARIZ in various engineering systems has been employed, but most of the cases did not cover all the requirements and processes of ARIZ.

In this research, acrylic polymer microspheres modified with succinimide functional groups via N-acryloxysuccinimide (NAS) moieties was used as the matrix for DNA probe immobilisation. As previously reported by Chen and Chiu 2000 and Chaix et al. 2003 [19, 20], the succinimide functional group can react with amine functional groups to form a covalent bond. The incorporation of NAS functionality into acrylic microspheres for DNA microbiosensor application provides advantages of a simple preparation method where the spheres can be synthesised and functionalised via a one-step procedure using photopolymerisation in a short duration (several minutes). In addition, the microspheres have the advantage of small size and provide a large surface area for DNA probe immobilisation, thus reducing the barrier to diffusion for reactants and products. This enables the improvement in the biosensor performance in terms of shorter response times and wider linear response range, which will be demonstrated in the work reported here.

In this study, an electrochemical DNA biosensor method, which is highly sensitive, simple, easy-to-fabricate, and low cost, is proposed for juvenile arowana fish gender determination with high accuracy. The DNA biosensor was built from a carbon screen printed electrode (SPE) modified with colloidal gold nanoparticles (AuNPs) and polyacrylate microspheres functionalised with NAS functional group. The AuNPs were immobilised onto the carbon SPE surface via electrostatistic interaction and played an important role in enhancing the electrode conductivity and facilitating the electron transfer, while the acrylic microspheres (AcMPs) were directly deposited onto the AuNP-modified SPE via physical adsorption. Aminated DNA probe of arowana was then covalently attached to the immobilised AcMP-AuNP composite at the exposed succinimide group of AcMPs. Probe-target hybridisation was detected with anthraquinone redox label via differential pulse voltammetry (DPV). The incorporation of small and uniform size of AcMPs was able to hold a large DNA-loading capacity and enhancing the sensitivity and detection limit of the electrochemical arowana DNA biosensor.

## Methods

### Apparatus and Electrodes

All the electrochemical measurements were performed with DPV using Autolab PGSTAT 12 potentiostat/galvanostat (Metrohm) at 0.02 V step potential within the potential window of −1.0 V to −0.1 V. SPE from Scrint Technology Co Malaysia modified with AcMPs and AuNPs was used as the working electrode. A rod-shaped platinum (Pt) electrode and an Ag/AgCl electrode filled with 3.0 M of KCl internal solution were used as auxiliary and reference electrodes, respectively. Elma S30H sonicator bath was used to prepare homogeneous solutions.

### Chemicals

2–2-Dimethoxy-2-phenylacetophenone (DMPP) was purchased from Fluka. 1,6-Hexanediol diacrylate (HDDA), n-butyl acrylate (nBA), and Au (III) chloride trihydrate were supplied by Sigma-Aldrich. The colloidal AuNPs was synthesised according to the method reported by Grabar et al. (1995). Sodium dodecyl sulphate (SDS) and NaCl were obtained from Systerm. NAS and anthraquinone-2-sulfonic acid monohydrate sodium salt (AQMS) were procured from Acros. Milli-Q water (18 mΩ) was used to prepare all the chemical and biological solutions. Stock solution of DNA probe was diluted with 0.05 M of K-phosphate buffer (pH 7.0) while complementary DNA (cDNA) and non-complementary (ncDNA) solutions were prepared with 0.05 M of Na-phosphate buffer at pH 7.0 containing 1.0 mM of AQMS. The K-phosphate buffer facilitates maximum DNA probe immobilisation on the succinimide-functionalised acrylic material, whereas the Na-phosphate buffer provides an optimum condition for DNA hybridisation reaction [21, 22].

### Synthesis of Acrylic Microsphere

AcMPs were prepared according to the methods described previously with slight modification [22]. Briefly, a mixture of 450 μL of HDDA, 0.01 g of SDS, 0.1 g of DMPP, 7 mL of nBA monomer, and 6 mg of NAS was dissolved into 15 mL of Milli-Q water and sonicated at room temperature (25 °C) for 10 min. After that, the emulsion solution was photocured with UV light for 600 s under a continuous flow of N_2_ gas. The resulting poly(nBA-NAS) microspheres were then collected by centrifugation at 4000 rpm for 30 min followed by washing in K-phosphate buffer (0.05 M, pH 7.0) for three times and left to dry at ambient temperature.

### Fabrication of DNA Biosensor Using Acrylic Microspheres

**Fig. 1:**
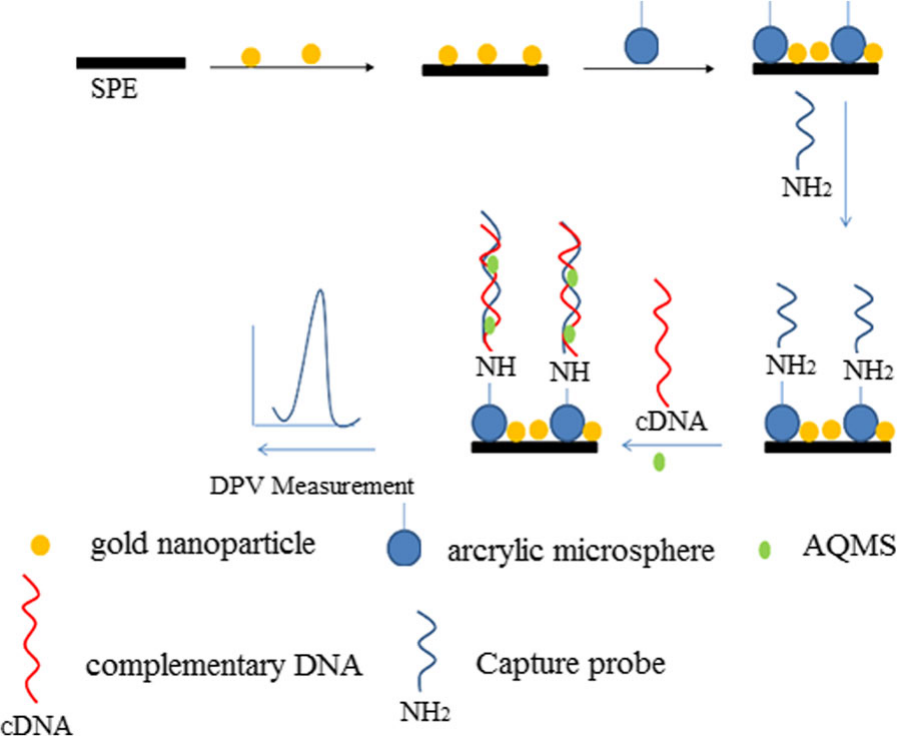
The fabrication procedure of electrochemical arowana DNA biosensor based on AcMP-AuNP-modified electrode

Prior to surface modification, the carbon SPE was rinsed thoroughly with DI water, drop-coated with the acrylic polymer microspheres at 3 mg/mL, and allowed to air-dry at ambient conditions, followed by drop-casting with 5 mg/mL of colloidal AuNPs. The electrochemical characteristic of carbon SPE before and after modification with AcMPs and AuNPs was examined with CV method. Figure 1 portrays the method, which is composed of 3-step fabrication of electrochemical DNA biosensor and 1-step arowana cDNA detection. About 10 μL of colloidal AuNPs (1 mg/300 μL) was firstly deposited onto a carbon SPE and air dried at 25 °C. As the AcMPs (1 mg) was readily suspended in ethanol (100 μL) to form a stable dispersion, 10 μL of AcMP suspension was drop-coated onto the AuNP-modified SPE. The AcMP-AuNP-modified carbon SPE was then dipped in 300 μL of 5 μM arowana DNA probe solution for 6 h for DNA immobilisation process to take place and washed carefully with K-phosphate buffer (0.05 M, pH 7.0) for three times to remove the unbound capture probe. The immobilised DNA probe was later immersed in 300 μL of target DNA solution containing 2 M of NaCl and 1 mM of AQMS to allow DNA hybridisation and intercalation reactions to occur within an hour, followed by sequentially rinsing with Milli-Q water and Na-phosphate buffer (0.05 M, pH 7.0) for the removal of non-hybridised DNA fragments and a specific binding of AQMS electrochemical label. All the DPV measurements were performed in 4.5 mL of 0.05 M of K-phosphate buffer at pH 7.0 and room temperature.

### Optimization of Electrochemical Arowana DNA Biosensor

The DNA electrodes modified with the respective AcMP, AuNP, and AcMP-AuNP composite were used in the cDNA (5 μM) and ncDNA (5 μM) testing with DPV electroanalytical method in the presence of 1 mM of AQMS and 2 M of NaCl at the scan rate of 0.5 V/s versus Ag/AgCl reference electrode. DNA probe immobilisation duration was determined by separately soaking nine units of AcMP-AuNP-modified SPEs in 300 μL of 5 μM arowana DNA probe solution for 1, 2, 3, 5, 6, 7, 8, 12, and 18 h, before reaction with 5 μM of cDNA in DNA hybridisation buffer (0.05 M of Na-phosphate buffer at pH 7.0) containing 1 mM of antraquinone redox intercalator and 2 M of NaCl. DNA hybridisation time was investigated by immersing the DNA electrode in 300 μL of 5 μM cDNA solution in the presence of 2 M of NaCl and 1 mM of AQMS for 10–100 min. The effect of temperature on the DNA hybridisation duration was done by measuring the arowana DNA biosensor response at 4, 25, 40, and 50 °C for an experimental period of 5–90 min in the measuring buffer using DPV technique. For pH effect study, the arowana DNA biosensor was dipped in 5 μM of cDNA solution prepared from 0.05 M of Na-phosphate buffer conditioned with 2 M of NaCl and 1 mM of AQMS between pH 5.5 and pH 8.0 followed by DPV measurement. The effect of various positively charged ions (i.e. Ca^2+^, Na^+^, K^+^, and Fe^3+^ ions) on the electrochemical arowana DNA biosensor response was carried out by adding CaCl_2_, NaCl, KCl, and FeCl_3_ into 0.05 M of Na-phosphate buffer (pH 7.0) prior to DNA hybridisation reaction and DPV measurement. Ionic strength of the hybridisation buffer was optimised by varying the Na-phosphate buffer and NaCl concentrations from 0.002–0.1000 M to 1.52–5.50 M, respectively. The linear calibration curve of the arowana DNA biosensor was then established through quantitative measurement of a series of cDNA concentrations from 1.0 × 10^−18^ to 2.0 × 10^−2^ μM via DPV method. All the experiments were performed in triplicate.

### DNA Extraction and Arowana DNA Analysis

A total of 15 arowana fish tissue samples were kindly provided by Fisheries Research Institute (FRI), Department of Fisheries Malaysia. All the fish tissue samples were stored in 70% ethanol in a chiller at 4 °C and dispatched to the laboratory. The fish tissue samples were washed with Milli-Q water and cut into small pieces and dried at ambient conditions before kept in the freezer at −20 °C. Arowana DNA from each tissue sample (35–40 mg each) was then separately extracted using QIAquick PCR Purification kit (Manchester, UK) according to the manufacturer’s protocol and stored at −20 °C when not in use. PCR amplification of genomic DNA fragment was then performed using Bio-Rad PCR thermal cycler (PTC-100, Hercules, USA). The DNA fragments of PCR product were then separated with 1.5% agarose gel electrophoresis. The arowana DNA extracts were also analysed by the electrochemical DNA biosensor to determine the gender. The DPV responses obtained were compared with the baseline current obtained without the presence of arowana DNA. A *t* test was applied to determine significant difference between the DNA biosensor response and baseline current at 4 degrees of freedom and 95% confidence level. The DNA biosensor response obtained at significantly higher than the baseline current indicated a male arowana fish was detected and vice versa.

## Results and Discussion

**Fig. 2:**
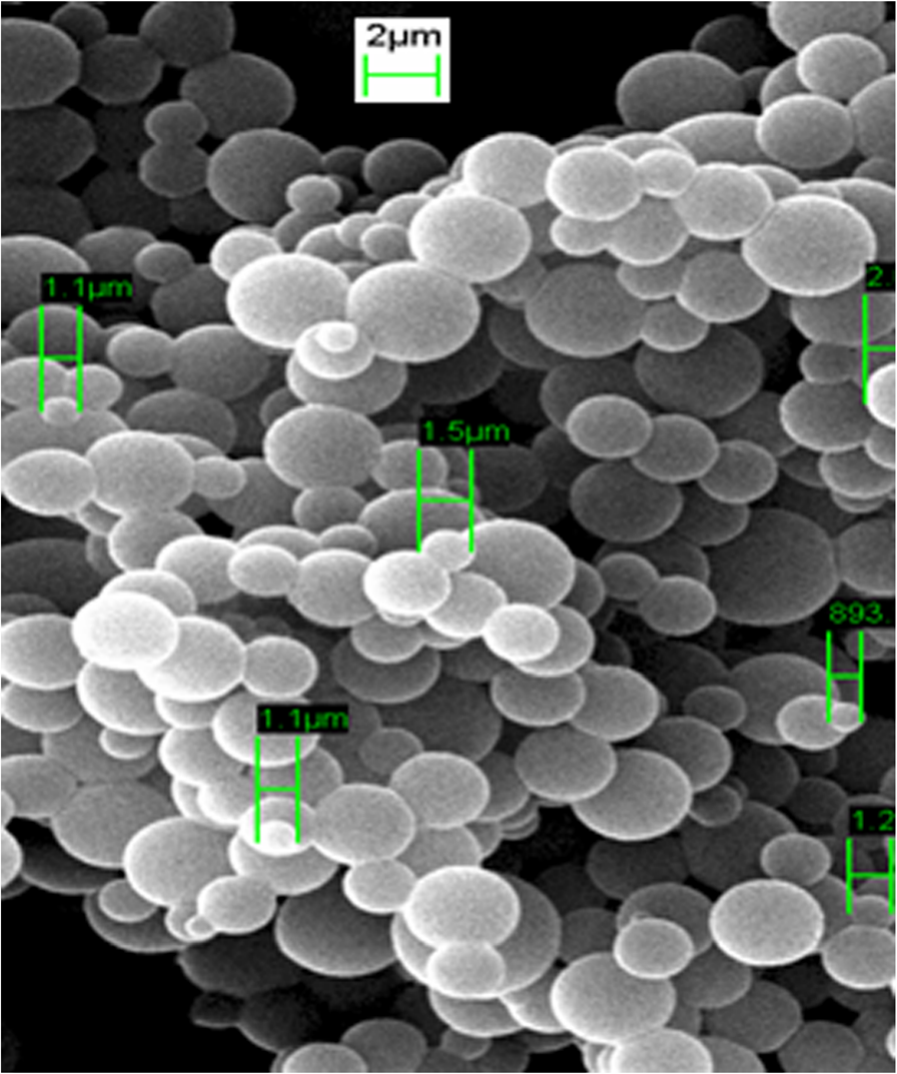
SEM image of acrylic polymer microspheres

**Fig. 3:**
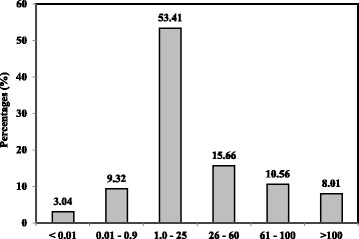
Size distribution of acrylic micropsheres prepared from photopolymerisation

The as-synthesised AcMPs were observed (Fig. 2) under scanning electron microscope (SEM, LEO 1450VP). The size distribution of acrylic miscropsheres prepared from photopolymersation is illustrated in Fig. 3.

**Fig. 4:**
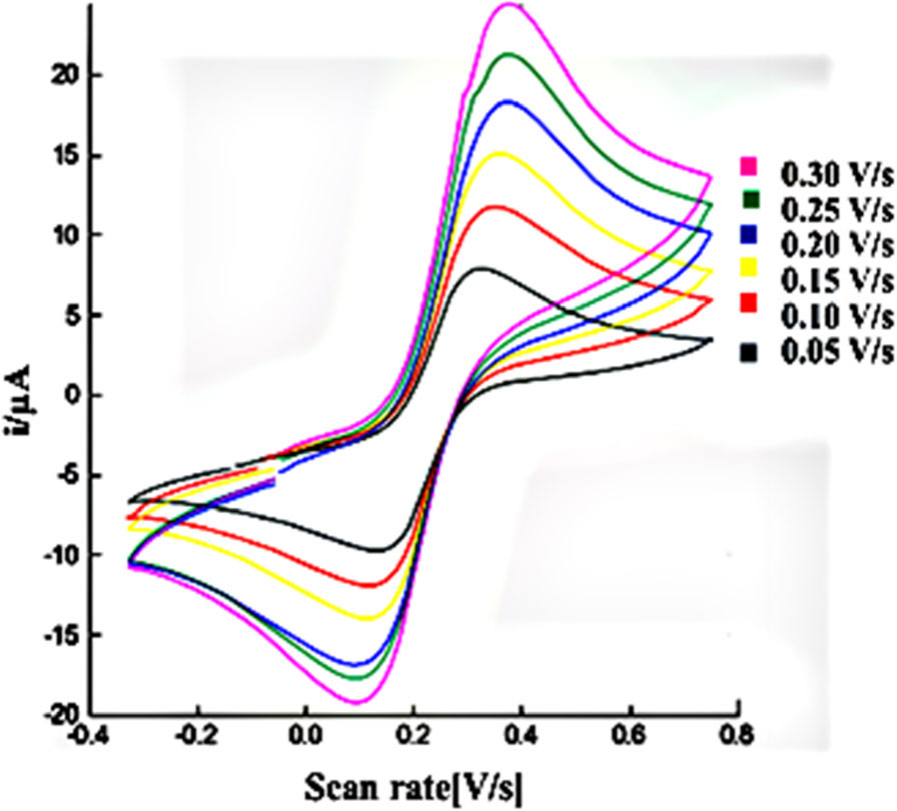
Cyclic voltammograms of 1.0 mM K_3_Fe(CN)_6_ in 0.05 M Na-phosphate buffer of pH 7.0 with different scan rates (0.05, 0.10, 0.15, 0.20, 0.25, and 0.30 V/s) for a modified carbon SPE containing AcMP-AuNP material at the electrode surface

The effect of the different scan rates of the carbon SPE containing AcMPs-AuNPs in the presence of K_3_Fe(CN)_6_ showed that the oxidation and reduction peak currents increased with the increasing of the scan rate from 0.05 to 0.30 V/s (Fig. 4). Thus, the electron transfer process at the electrode surface is expected to be reversible [22–25].

**Fig. 5:**
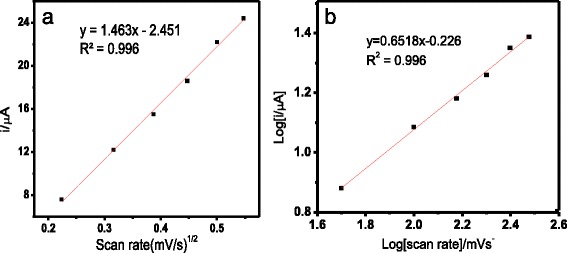
Plot of the oxidation peak currents (ip/μA) versus square root of scan rate ((mV/s)^1/2^) (**a**) and plot of log of oxidation peak currents (ip/μA) versus log of scan rates (log (mV/s)) (**b**)

Based on the Randles–Sevcik equation,

1 $$ \mathrm{ip}=0.4463\ \mathrm{nFAC}\ {\left(\mathrm{nFvD}/\mathrm{RT}\right)}^{1/2} $$

a good linearity was found between the redox peak current and the square root of the scan rate with a correlation coefficient (*R* ^2^) of 0.996 within the range of 50–300 mV/s as shown by Eq. 2 and Fig. 5a.

2 $$ \mathrm{ip}=1.463{\mathrm{v}}^{1/2\hbox{--} }2.451 $$

This indicates that the reaction at the surface of the modified electrode was a diffusion controlled reaction [22–25].

Furthermore, based on Fig. 5b, when the log value of oxidation current was plotted against the log value of scan rate, a linear line was obtained with a slope of 0.65, which was close to the theoretical value of 0.50 for diffusion-controlled process. Therefore, the study has demonstrated that the reaction at the surface of the modified SPE is mostly diffusion controlled.

For the ideal case of a fast, reversible, and one-electron transfer process, ΔEp = 0.059 V at 298 K. However, the peak potential shifts that increased with the scan rate demonstrated larger peak potential separations of more than 0.059 V (Fig 4). This implies that the electron transfer process at the electrode surface is slow [22, 25, 26], probably due to the resistance created by the presence of AcMP material covering the electrode surface.

**Fig. 6:**
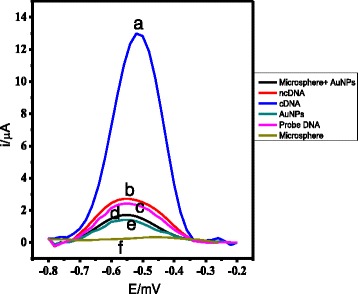
The DPV signal of AcMP-AuNP-based DNA electrode upon hybridisation with cDNA (**a**) and non-complementary DNA (**b**), the DPV response of the AcMPs (**f**) and AuNP-modified SPE (**e**), and AcMP-AuNP composite modified SPE as well as the response of DNA biosensor based on AcMP-AuNP composite modified probe DNA SPE (**c**) before reaction with cDNA in the presence of 1 mM AQMS at the scan rate of 0.5 V/s versus Ag/AgCl reference electrode

Figure 6 shows the DPV response of arowana DNA biosensor based on AcMP, AuNP, and AcMP-AuNP-modified carbon SPEs. The significant DPV current difference observed between experiment (a) and (c) reveals that the arowana DNA probes were successfully grafted onto the AcMPs via strong covalent bonds between succinimide functional group of AcMP and amine functional group of the aminated DNA probe, and the immobilised arowana DNA probe was selective only to its cDNA [19, 20]. The AuNPs played a role to assist the electron conductivity from the intercalated AQMS to the fabricated electrode surface. Without the inclusion of AuNPs in the composite material (f), only gold nanoparticles (e), and the gold nanoparticles and AcMP composite (d), only very little current response can be observed. The low DPV currents acquired in experiment (b) was due to no DNA hybridisation reaction occurred with ncDNA, which also indicates no specific absorptions of AQMS redox indicator on the electrode surface [27, 28].

**Fig. 7:**
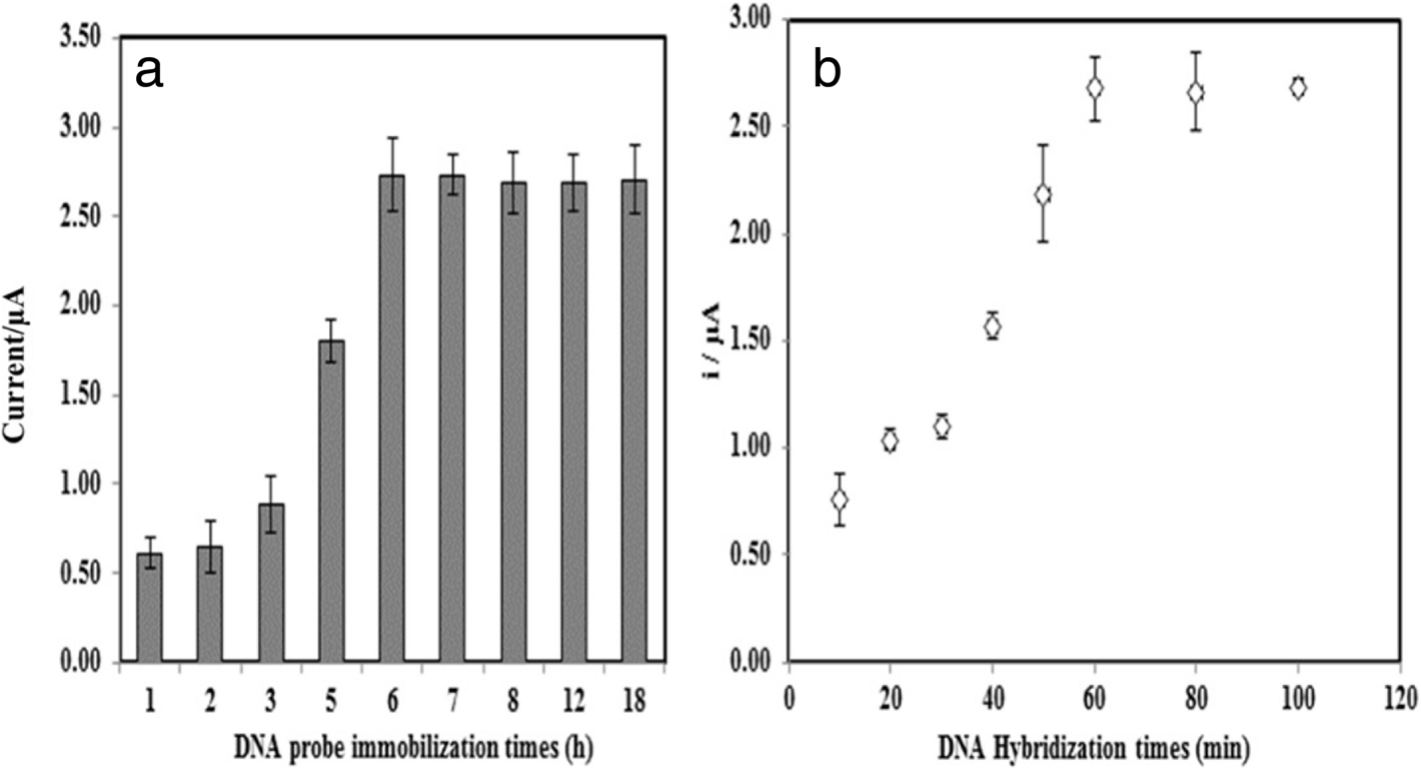
Effects of DNA probe immobilisation time (**a**) and DNA hybridization time (**b**) on the arowana DNA biosensor response using 5 μM DNA probe and cDNA in the presence of 1 mM AQMS at 2 M ionic strength

For DNA probe immobilisation duration, Fig. 7a exhibits the DNA biosensor response slowly increased over the first 1–3 h of DNA probe immobilisation time and the abrupt increase in the DNA biosensor response can be seen between 3 and 6 h of DNA probe immobilisation duration. This was because a longer immobilisation time was required to promote larger amount of DNA probes to be attached on the AcMP-AuNP-modified electrode. At a further prolonging of DNA probe immobilisation time, no noticeable change in the DNA biosensor response was perceived as the binding sites of immobilised AcMPs have fully bound with DNA probes. The arowana DNA biosensor response is also dependent on the DNA hybridisation time. The biosensor response profile illustrated in Fig. 7b shows an increasing DPV current response trend with DNA hybridisation duration from 10 to 60 min, after which the current response becomes almost plateau. At this stage, the immobilised DNA probes on the electrode have entirely hybridised with cDNA [29].

**Fig. 8:**
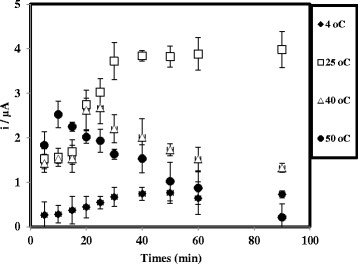
Effect of temperature on the DNA hybridisation time of arowana DNA biosensor. The DPV response was measured in 0.05 M K-phosphate buffer (pH 7.0) at 4, 25, 40, and 50 °C for an experimental period of 5–90 min

It is also noticed that the DNA hybridisation time of the fabricated arowana DNA biosensor was temperature dependent, and as a great advantage, we obtained a maximum current response at room temperature within 30 min (Fig. 8). At low temperature, i.e. 4 °C, a long time was required for a complete DNA hybridisation reaction because the cold temperature slowed down the DNA hybridisation reaction rate. A faster DNA hybridisation time could be achieved at a temperature above 25 °C ascribed to the higher DNA hybridisation reaction rate occurred between immobilised DNA probe and cDNA to form the duplex DNA at high temperatures. However, high temperature could permanently deform the double-helical structure of DNA, and regeneration of the DNA molecule is not possible even after the readjustment of the temperature to the optimal value [28, 30].

**Fig. 9:**
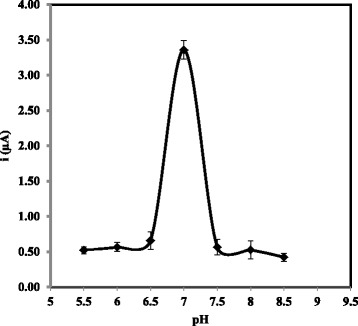
The DPV response of arowana DNA biosensor based on AcMP-AuNP composite modified carbon SPE between pH 5.5 and pH 8.0. The DPV measurement was conducted in 0.05 M K-phosphate buffer (pH 7.0) at 25 °C and scan rate of 0.5 V/s versus Ag/AgCl reference electrode

As part of the arowana DNA biosensor response optimisation, the effect of solution pH on the DNA hybridisation reaction was investigated. The DNA biosensor showed negligible current change between pH 5.5 and pH 6.5 due to the protonation of phosphodiester backbone of DNA, which reduced the solubility of DNA molecules in aqueous environment (Fig. 9). Further increase in pH of the DNA hybridisation medium, the arowana DNA biosensor response increased abruptly at pH 7.0, after which a sharp decline in DPV current was discernible as the pH environment changed to basic condition due to the irreversible denaturation of DNA in the higher pH range [23, 24, 31–33]. Since maximum DPV response was acquired at a neutral pH, the next electrochemical evaluation of arowana DNA biosensor response was maintained at pH 7.0 using 0.05 M of Na-phosphate buffer.

**Fig. 10:**
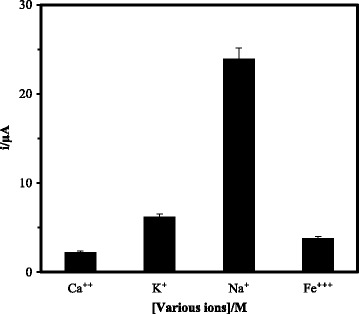
The effect of Ca^2+^, Na^+^, K^+^, and Fe^3+^ ions in the DNA hybridisation buffer (0.05 M Na-phosphate buffer at pH 7.0) on the DPV response of arowana DNA biosensor

The effect of valency of cations towards DNA hybridisation reaction was performed using different cations of salts, e.g. Ca^2+^, Na^+^, K^+^, and Fe^3+^ ions in the DNA hybridisation buffer. The positively charged ions could interact electrostatically with the negatively charged phosphodiester chain of DNA molecule to overcome the steric hindrance and electrostatic repulsion between the immobilised DNA probe and target DNA, thereby facilitates the DNA hybridisation process [34]. Figure 10 demonstrates that the DNA hybridisation reaction was favourable in the presence of cations in the order of Na^+^ > K^+^ > Fe^3+^ > Ca^2+^. The presence of Ca^2+^ and Fe^3+^ ions were noticed to cause a remarkable decrement in the arowana DNA biosensor current response compared to Na^+^ and K^+^ ions. These phenomena were attributed to the formation of sparingly soluble calcium phosphate and ferrum (III) phosphate salts in the DNA hybridisation buffer [22], which reduced the ionic content of the solution and caused a high electrostatic repulsion between the DNA molecules. As a result, the DNA hybridisation rate was declined and led to a poor biosensor performance. The highest DNA biosensor response was obtained when Na^+^ ions were added to the DNA hybridisation phosphate buffer because of their small size and strong affinity towards the DNA phosphodieter bond.

**Fig. 11:**
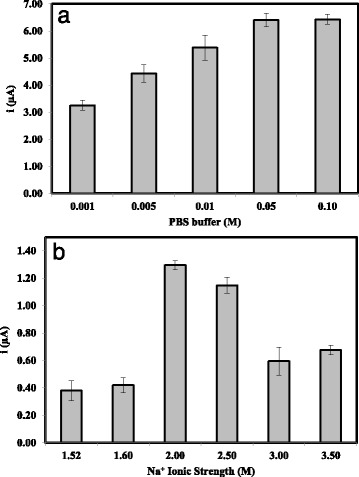
The arowana DNA biosensor response trends as the **a** Na-phosphate buffer concentration and **b** ionic strength of the hybridisation buffer varied from 0.002–0.100 M and 1.52–5.50 M, respectively

The concentration of NaCl and Na-phosphate buffer (pH 7.0) must also be optimised to provide an optimal ionic strength for hybridisation buffer. Figure 11b indicates that ionic strength of below and above 2 M could not overcome the high electrostatic repulsion between DNA strands. About 0.05 M of Na-phosphate buffer (Fig. 11a) and 2 M of NaCl were found to provide the optimum ionic strength for the assay of arowana target DNA with maximum biosensor performance. Optimum hybridisation buffer conditions in terms of pH, buffer capacity, and ionic strength would allow DNA hybridisation reaction to occur at the most minimum steric hindrance [30].

**Fig. 12:**
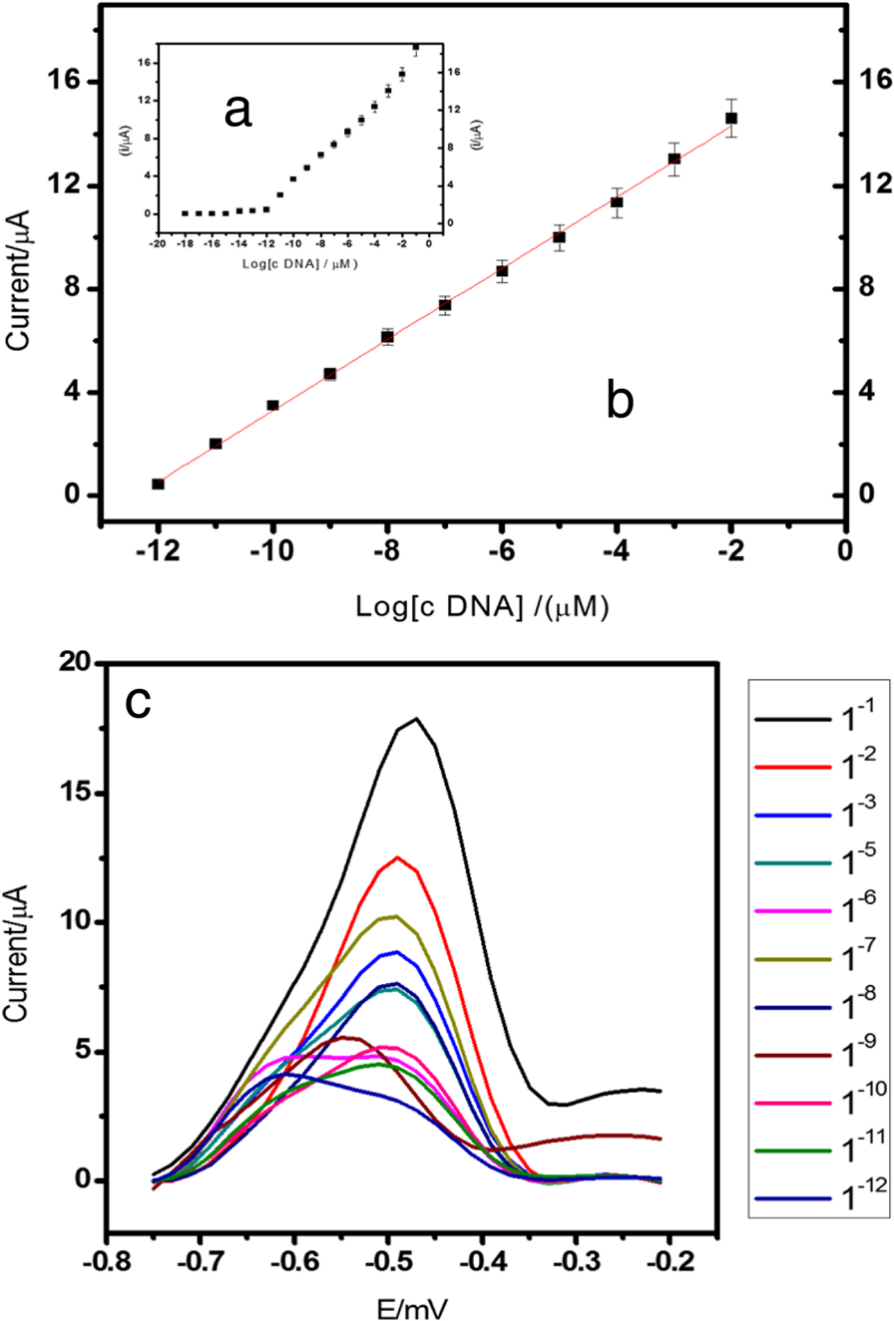
The arowana DNA biosensor response curve (**a**) and linear calibration range (**b**) and the DPV voltammogram (**c**) obtained using 1.0 × 10^−18^ to 1.0 × 10^−2^ μM cDNA at pH 7.0

The optimised DNA biosensor was then used for the detection of a series of arowana cDNA concentrations between 1.0 × 10^−12^ and 1.0 × 10^−2^ μM. The DNA biosensor showed a wide linear response range from 1.0 × 10^−18^ to 1.0 × 10^−8^ M (*R* ^2^ = 0.99). The limit of detection (LOD) obtained at 1.0 × 10^−18^ M was calculated based on three times the standard deviation of biosensor response at the response curve approximating LOD divided by the linear calibration slope. The homogeneous AcMP particles size within micrometre range exhibited a significant influence on the DNA biosensor sensitivity and reproducibility (RSD = 5.6%). The large binding surface area of the immobilised NAS-functionalised AcMPs permitted a large number of DNA molecules to bind covalently to the electrode surface, thereby increasing the DNA biosensor analytical performance with respect to dynamic linear range and detection limit of the arowana DNA biosensor (Fig. 12).

### Determination of Arowana Fish Gender with DNA Biosensor

The developed electrochemical DNA biosensor has been validated with the standard PCR-based method to determine the gender of Asian arowana fish. With the results tabulated in Table 2, both methods provided the same result for the gender determination of arowana fish. This indicates that the proposed DNA biosensor can be used for accurate determination of arowana gender in a simple and fast way.

**Table 1:** Sequences of oligonucleotides utilised in the present investigation

DNA	Base sequences
DNA Probe	5'-AAT TCA AGG GAA CTG ATG ACT CTA (AmC7)
cDNA	5'-TAG AGT CAT CAG TTC CCT TGA ATT
ncDNA	5'-CGA GCG ACG TGA GCT TAG CTG CGC

**Table 2:** A comparison between DNA biosensor and PCR method in the gender identification of arowana fish using fish tissue samples

No	Sample	DNA biosensor method					PCR method
		Current (μA)	RSD	Baseline ± SD	*t* test	Gender	
1	227	1.479 ± 0.138	9.360	1.885 ± 0.10	5.042**^X^	F	F
2	231	2.315 ± 0.149	6.453	1.885 ± 0.10	7.184**^Y^	M	M
3	232	2.627 ± 0.185	7.053	1.885 ± 0.10	7.219**^Y^	M	M
4	233	1.829 ± 0.158	8.643	1.885 ± 0.10	1.117	F	F
5	236	2.021 ± 0.169	8.372	1.885 ± 0.10	1.387	F	F
6	417	2.947 ± 0.215	7.291	1.956 ± 0.06	10.412**^Y^	M	M
7	437	2.779 ± 0.089	3.217	1.956 ± 0.06	22.126**^Y^	M	M
8	450	1.964 ± 0.122	6.215	1.956 ± 0.06	0.093	F	F
9	530	2.500 ± 0.232	9.264	1.956 ± 0.06	4.542**^Y^	M	M
10	531	2.581 ± 0.195	7.556	1.956 ± 0.06	6.544**^Y^	M	M
11	537	2.001 ± 0.189	9.441	1.993 ± 0.12	0.124	F	F
12	521	1.672 ± 0.043	2.600	1.993 ± 0.12	6.599**^X^	F	F
13	524	2.774 ± 0.102	3.678	1.993 ± 0.12	10.775**^Y^	M	M
14	525	1.359 ± 0.075	5.512	1.993 ± 0.12	7.377**^X^	F	F
15	526	2.953 ± 0.169	5.731	1.993 ± 0.12	10.857**^Y^	M	M

*M* male, *F* female

**^Y^–DPV current significantly higher than the baseline current (obtained from PBS buffer alone) indicates male fish; **^X^–DPV current significantly lower than the baseline current indicates female fish, critical value t_4_ = 2.78 (*p* = 0.05, 95%)

## Conclusions

The electrochemical DNA biosensor developed in this study demonstrated good sensitivity, wide linear response ranges, and low detection limit in the determination of arowana target DNA. In addition, the DNA biosensor showed a good response towards arowana cDNA, which implies that the electrochemical DNA biosensor could be used to successfully detect the arowana DNA segments. The developed arowana DNA biosensor can be further redesigned into a point-of-use device prototype that offers a great potential for the application in the fish culture for early identification of arowana gender and colour, which is economically advantageous in fishery and aquaculture sectors.
